# Application of a naturalistic psychogenic stressor in periadolescent mice: effect on serum corticosterone levels differs by strain but not sex

**DOI:** 10.1186/1756-0500-3-170

**Published:** 2010-06-17

**Authors:** Christine H Kapelewski, Jeanette M Bennett, Sonia A Cavigelli, Laura C Klein

**Affiliations:** 1 Biobehavioral Health Department, 315 East Health and Human Development Building, The Pennsylvania State University University Park, PA, USA

## Abstract

**Background:**

As a first step in determining whether psychogenic stressors might be incorporated into periadolescent mouse models of stress, we evaluated whether a commonly used psychogenic stressor, exposure to red fox urine, alters serum corticosterone levels in periadolescent C57BL/6J and DBA/2J mice.

**Findings:**

In a 1-day experiment, forty-eight 38-day-old C57BL/6J (N = 12 males; N = 12 females) and DBA/2J (N = 12 males; N = 12 females) mice were exposed to 10-min of red fox urine via cotton ball (N = 12 C57BL/6J mice; N = 12 DBA/2J mice) or to a non-saturated cotton ball (N = 12 C57BL/6J mice; N = 12 DBA/2J mice). All mice were sacrificed 15-min after cotton ball exposure and serum was collected for corticosterone assessment. Overall, there was a main effect for strain such that C57BL/6J male and female mice displayed higher corticosterone levels than did male and female DBA/2J mice. There were no main effects for sex or odor exposure. However, there was a significant strain by odor exposure interaction, whereby, within odor-exposed mice, DBA/2J mice displayed lower corticosterone levels (ng/mL) compared to C57BL/6J mice, regardless of sex. Further, among DBA/2J mice, predator odor exposure reduced corticosterone levels compared to no odor exposure.

**Conclusions:**

Findings indicate that mouse strain, but not sex, may play an important role in the efficacy of a predator odor among periadolescent mice.

## Background

The majority of rodent stress models use physical stressors, such as footshock, to elicit the classic hypothalamic-pituitary-adrenal (HPA) axis stress response that results in elevated corticosterone levels. Although physical stressors are effective in eliciting these neuroendocrine responses, many of these stressors have the potential confound of pain. In contrast, psychogenic stressors (e.g., predator odor) are effective in inducing similar behavioral and glucocorticoid responses without causing pain [1]. Excretions or scents of predators (e.g., fox, cat, wolf, coyote) reliably signal danger to rodents and exposure to these compounds results in significant neuroendocrine [2-4] and behavioral (e.g., freezing; rearing and/or locomotion; digging) [2-7] changes indicative of stress. Importantly, these effects are observed in predator odor-naïve rodents; animals display anxiety-like behaviors and corticosterone responses following exposure to predator odor [1,5,8]. Several reviews [8-10] and elegant studies have been published in this area examining multiple types of predator exposure, ranging from the presence of the actual predator (e.g., cat) [5] to the predator's excretions (e.g., urine, feces) [7] to exposure to 2,3,5-trimethyl-3-thiazoline (TMT), a synthetic form of the purported fear-inducing component of fox fecal matter [9,11,12]. Given this prior work on naturalistic psychogenic stressors with adult rodents, in the present study, we examined the extent to which predator odors elicit physiological stress in periadolescent rodents (mice in particular).

There is increased interest in bridging adult rodent models of stress with periadolescent models in order to understand developmental influences of stress on health outcomes such as anxiety and depression in humans [13,14]. With the exception of two recent studies on the biobehavioral effects of cat odor stress in juvenile and periadolescent Long-Evans rats [4,15], no other literature is available on the effectiveness of predator odor stressors among periadolescent rodents. We were particularly interested in bridging this work into a mouse model because mouse models are essential for determining genetic contributions to stress responses and health; periadolescent mouse models are useful for elucidating developmental mechanisms involved in individual differences in stressor vulnerability during an established sensitive period of life. Thus, as a first step in determining whether psychogenic stressors might be incorporated into periadolescent mouse models of stress, we evaluated whether a commonly used psychogenic stressor, exposure to red fox urine, alters serum corticosterone levels in periadolescent mice. Two common inbred strains of mice, C57BL/6J and DBA/2J, were selected for their use in adult biobehavioral stress studies [16,17] as well as for their differential sensitivity to stress as adults [17,18]. Our mice were periadolescent [post natal day (PN) 38] at testing to align with the periadolescent time frame according to Spear [19]. Male and female mice were included in order to examine any sex differences in corticosterone responses to predator odor exposure.

To the best of our knowledge, this is the first examination of a predator odor stressor in periadolescent male and female mice. Taking into account established and published procedures with adult rats [6], we hypothesized that exposure to red fox urine for 10 minutes would result in higher corticosterone levels in exposed periadolescent mice compared to non-exposed mice, and that DBA/2J mice would display higher corticosterone levels compared to their C57BL/6J counterparts, regardless of odor exposure [18]. We did not hypothesize a sex difference in the effects of a fox urine odor exposure on corticosterone due to a lack of literature in this area.

## Methods

### Animals

Forty-eight, 30-day-old male and female C57BL/6J (N = 24; 12 males, 12 females) and DBA/2J (N = 24; 12 males, 12 females) mice (*Mus musculus*; Jackson Laboratories, Bar Harbor, ME, USA) were individually housed in conventional Plexiglas shoebox cages without filter tops, filled with ¼ inch bedding (Bed-o'Cobs, The Andersons Agriservices, Inc., Manmee, IL, USA). Mice were periadolescent (36-38 days of age) [19-22] during the baseline and predator odor exposure phases of the experiment. Mice were kept in climate-controlled rooms, with a temperature of 21 ± 2°C, a relative humidity of 41%, and maintained on a 12/12-h light-dark cycle (lights on at 0700). Throughout the study, mice had continuous access to standard rodent chow (Lab Diet 5001 Rodent Diet, PMI Nutrition International, Brentwood, MO, USA) and tap water. The Pennsylvania State University Institutional Animal Care and Use Committee reviewed and approved all animal use procedures (IACUC Protocol #30411), all of which followed previously established guidelines [23].

### Experimental Procedure

#### Acclimation (5 days)

Following arrival at 30 days of age, mice were left undisturbed in their home cages for 5 days to acclimate to the animal housing facility.

#### Baseline (2 days)

At 36 days of age (PN36), mice were weighed, and food and water consumption were measured for two consecutive days. These measurements were used to assign mice to control (n = 24; 12 C57BL/6J, 12 DBA/2J) or predator odor treatment (n = 24; 12 C57BL/6J, 12 DBA/2J) groups to ensure that control and odor groups did not differ significantly in body weight, food or water intake.

#### Predator Odor Exposure (1 day)

At 38 days of age (PN38), mice weighed on average 16.61 ± 0.33 g. Following body weight, food and water intake measurements (0800 hrs), mice in the odor-exposure condition (C57BL/6J: 6 males, 6 females; DBA/2J: 6 males, 6 females) were transferred to the treatment room (several corridors away), allowed to acclimate for 15 mins, and then exposed to a fresh cotton ball saturated with 1 mL of red fox urine (Buck Stop Lure Company, Stanton, MI, USA) in their home cages for 10 mins in a fume hood. Each cotton ball was covered by a plastic cup with 30 holes punched in it, allowing the scent to permeate the cage while preventing mice from coming into direct contact with the cotton ball. In the housing room, control mice were placed in a fume hood in their home cages with a non-saturated cotton ball covered by a plastic cup that also contained 30 holes. To prevent odor contamination, experimenters administering the fox odor exposure had no contact with experimenters handling control mice in the housing room. In addition, each mouse received a fresh cotton ball and cup.

### Serum Corticosterone Assessment

Fifteen minutes after the end of cotton ball exposure, mice were sacrificed via cervical dislocation. Sacrifice began 2 hours into the light cycle (0900 hrs) at the nadir of the diurnal corticosterone rhythm to maximize the influence of stress on HPA-axis activity. Sacrifice order was counterbalanced across sex, strain, and odor group. Individuals performing necropsy had no exposure to fox urine animals until sacrifice. Trunk blood was collected and allowed to sit at room temperature for 15 minutes. Samples were then centrifuged at 1500 X g for 15 minutes and frozen at -80°C for later assessment of serum corticosterone. Corticosterone was determined by enzyme immunosorbent assay (EIA; Assay Designs, Ann Arbor, MI, USA) in the Behavioral Neuroimmunomodulation Laboratory at Penn State. Samples from each mouse were tested in duplicate in a single assay batch. Values used in data analyses are the averages of duplicate tests. The assay has a lower limit of sensitivity of 0.03 ng/mL, with an average inter- and intra-assay covariance (%) of less than 10% and 5%, respectively. The assay sensitivity (0.03 ng/mL) is based on the minimum corticosterone concentration required to produce a three standard deviation from assay A_0_.

### Statistical Analyses

**Figure 1:**
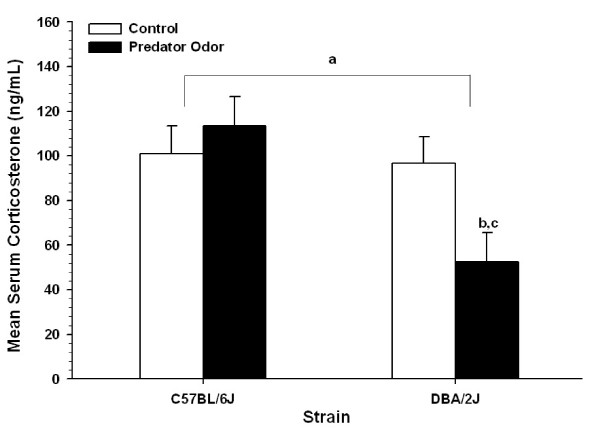
**Serum corticosterone levels (ng/mL) among C57BL/6J (N = 23) and DBA/2J (N = 24) control (□; N = 24) or predator odor-exposed (■; N = 23) mice (adjusted means + standard error of the mean)**. ^a^Strain effect (DBA/2J < C57BL/6J, p < 0.05); ^b^Strain X Odor Exposure interaction (Odor-exposed DBA/2J < Odor-exposed C57BL/6J, p < 0.05); ^c^Strain X Odor Exposure interaction (Odor-exposed DBA/2J < Control DBA/2J, p < 0.05).

An inadequate amount of blood was collected from an odor-exposed female C57BL/6J mouse for the assessment of corticosterone, and, therefore, was not included in corticosterone analyses. Because of rapid body weight gains during the periadolescent period [20,21], 3-way (sex X strain X stress) analysis of variance (ANOVA) was used to examine group differences in body weight on the last day of the experiment. There was a statistically significant strain X stress group interaction prior to stressor exposure. Therefore, body weight on the last day was included as a covariate in the following analyses (ANCOVA). Separate one-way ANCOVAs were used to examine statistically significant interactions. ANCOVA estimated marginal means adjusted for the body weight covariate (+ SEM) are reported in the corticosterone results section and Figure 1. All tests were two-tailed and statistical significance was set at alpha = 0.05.

## Results

### Body Weight

Table 1 presents mean body weights among each experimental group in the morning prior to predator odor or no-odor exposure. As expected, male mice weighed more than did female mice [F(1,47) = 15.03, p < 0.05] and C57BL/6J mice weighed more than did DBA/2J mice [F(1,47) = 28.08, p < 0.05]. Surprisingly, there was a sex X strain interaction in body weight [F(1,47) = 7.45, p < 0.05]. Among C57BL/6J mice, males weighed more than did female mice [F(1,23) = 40.42, p < 0.05], whereas males and females did not differ in body weight among DBA/2J mice. Among male mice, C57BL/6J mice weighed more than did DBA/2J mice [F(1,23) = 31.28, p < 0.05]. However, females weighed similarly across the two strains of mice (see Table 1).

**Table 1:** Mean body weight (g) among control and predator odor-exposed male and female periadolescent C57BL/6J and DBA/2J mice on predator exposure/no exposure treatment day.

	Control		Predator Odor-Exposed	
	Male (N = 12)	Female (N = 12)	Male (N = 12)	Female (N = 12)
**Strain**				
**C57BL/6J**	19.54 ± 0.43	16.24 ± 0.37^a,c^	19.29 ± 0.61	16.37 ± 0.52^a,c^
**DBA/2J**	15.80 ± 0.68^b,d^	15.70 ± 0.84^a,b^	15.46 ± 0.90^b,d^	14.48 ± 0.79^a,b^
				

^a^Sex effect (Females < Males), p < 0.05

^b^Strain effect (DBA/2J < C57BL/6J), p < 0.05

^c^Sex X Strain interaction (C57BL/6J female < C57BL/6J male), p < 0.05

^d^Sex X Strain interaction (DBA/2J male < C57BL/6J male), p < 0.05

Means ± standard error of the mean

### Corticosterone

Figure 1 presents serum corticosterone levels (ng/mL) for odor-exposed and control C57BL/6J and DBA/2J mice, collapsed across sex. Male and female mice displayed similar corticosterone levels among both strains and there was no main effect for predator odor exposure. However, there was a main effect for strain such that C57BL/6J mice displayed higher corticosterone levels compared to DBA/2J mice [F(1,38) = 4.61, p < 0.05] (see Figure 1). There also was a statistically significant strain X odor exposure interaction [F(1,38) = 5.87, p < 0.05] (see Figure 1). Specifically, C57BL/6J and DBA/2J mice in the control group had similar corticosterone levels, whereas in response to the predator odor DBA/2J mice displayed lower serum corticosterone levels compared to C57BL/6J mice [F(1,20) = 16.22; p < 0.05]. Among DBA/2J mice, predator odor exposure resulted in significantly lower serum corticosterone levels compared to no odor exposure [F(1,21) = 6.20; p < 0.05]. In contrast, odor exposure among C57BL/6J mice did not alter serum corticosterone levels. There were no other statistically significant 2- or 3-way interactions.

## Conclusions

To our knowledge, this is the first report on the effects of predator odor exposure in periadolescent male and female mice. Our data suggest that, during periadolescence, a single acute exposure to red fox urine decreases corticosterone levels among DBA/2J mice but does not change corticosterone levels among C57BL/6J, regardless of sex. Specifically, there was a strain by odor exposure interaction such that, among mice exposed to predator odor, DBA/2J mice displayed lower corticosterone levels compared to C57BL/6J mice. This reduced corticosterone response to red fox urine among DBA/2J mice, and no response among C57BL/6J mice is surprising in light of results with adult DBA/2J and C57BL/6J mice which demonstrate a strong corticosterone response to predator odor [5]. It is possible that a single exposure to predator odor was not sufficient to elicit a corticosterone response among periadolescent C57BL/6J mice. Alternatively, the strain difference in corticosterone responses may reflect differences in behavioral responses to stressors or novel situations in DBA/2J and C57BL/6J mice. For example, adult DBA/2J mice are more behaviorally proactive than are C57BL/6J mice when tested in response to a noxious non-predator odor and in a forced swim test; they spend more time actively burying a novel odor stimulus and less time immobile in the forced swim test compared to C57BL/6J mice [24,25]. This proactive response style may explain their lowered corticosterone response to the fox urine odor compared to the C57BL/6J mice [26,27]. Our opportunistic observations of the DBA/2J and C57BL/6J behavioral responses to the fox odor stimulus support this notion of differential response styles between the strains - adolescent DBA/2J mice actively engaged the fox odor presentation stimulus by climbing on top of the cup, whereas the C57BL/6J mice withdrew from the stimulus. This possible explanation for our observed strain differences requires further investigation; however, our strong strain difference in periadolescent mouse corticosterone responses to a predator odor stimulus is important background information for investigators interested in using psychogenic stressors with periadolescent mouse models. Additional investigations that include repeated odor exposure across the periadolescent window are needed to better understand the strain-specific effects of predator odor stressors during periadolescence. Jones and colleagues [18] reported complex strain and sex differences in bioadaptation (e.g., thymus weight, adrenal weight, corticosteroid binding globulin) to repeated immobilization stressor exposure in C57BL/6 and DBA/2 adult mice that were not present following a single stressor exposure. Their findings suggest that, for adults, full biological manifestations of stress may not become apparent until after several presentations of a stressor. It is important to determine whether repeated stressor exposure will have similar bioadaptation change, including corticosterone levels, in adolescents and how these changes translate into adulthood.

Interestingly, these two strains do not appear to differ in basal corticosterone levels, at least during adulthood [18,28]. This finding is consistent with the lack of a strain difference in corticosterone levels among our control periadolescent mice. Furthermore, our corticosterone levels among periadolescent control mice were similar to those previously reported with adults [18,28]. However, the inclusion of baseline corticosterone levels and alternative control conditions, such as non-predatory or novel odor cues (e.g., banana, strawberry, lemon), may help clarify this strain-specific response to a psychogenic stressor among periadolescent mice [4,5].

With regard to sex, males and females displayed similar corticosterone responses to the predator odor exposure, which is consistent with the lack of sex differences in behavioral responses to cat odor exposure among periadolescent (PN38) Long-Evans rats [15]. It seems that female, but not male, corticosterone sensitivity to cat odor exposure does not appear until later adolescence (PN47) and following repeated odor exposure [4]. Thus, it could be that the age, type of odor, and use of an acute exposure paradigm limited our ability to reveal any potential sex differences. Future studies should include repeated odor exposure with predator and non-predator odors and multiple ages in order to fully examine sex differences in corticosterone responses to predator odor stressors in periadolescent mice. The inclusion of outcome measures associated with defensive behavioral responses to predator odors (e.g., locomotion, freezing) and additional biological correlates of fear (e.g., dopamine receptor expression in the striatum and prefrontal cortex) are needed to extend prior studies with periadolescent rats into mice. Taken together, these data importantly suggest that the efficacy of predator odor stressors in inbred mice may vary across strains and across the lifespan.

## Abbreviations

HPA: Hypothalamic-pituitary-adrenal axis; TMT: 2,3,5-trimethyl-3-thiazoline; PN: post natal day; EIA: enzyme immunosorbent assay; ANOVA: analysis of variance; ANCOVA: analysis of covariance

## Competing interests

The authors declare that they have no competing interests.

## Authors' contributions

LCK provided funding. CHK, LCK, and JMB designed and performed the experiment. CHK drafted the introduction and methods sections. CHK and LCK analyzed the data and drafted the results section. SAC made intellectual contributions to the introduction and study design, and helped draft the discussion section. All authors edited all sections of the manuscript and approved this version of the submitted manuscript.
